# Delivering routine immunisations in London during the COVID-19 pandemic: lessons for future vaccine delivery. A mixed-methods study

**DOI:** 10.3399/BJGPO.2021.0021

**Published:** 2021-06-23

**Authors:** Helen Skirrow, Charlotte Flynn, Abigail Heller, Catherine Heffernan, Sandra Mounier-Jack, Tracey Chantler

**Affiliations:** 1 Department of Primary Care and Public Health, School of Public Health, Imperial College London, London, UK; 2 NHS England/Improvement (London Region), London, UK; 3 Public Health England based at NHS England/Improvement (London Region), London, UK; 4 Department of Global Health & Development, Faculty of Public Health & Policy, London School of Hygiene & Tropical Medicine, London, UK

**Keywords:** primary health care, general practice, health services, immunisation, COVID-19, COVID-19 vaccines

## Abstract

**Background:**

General practices in England have continued to care for patients throughout the COVID-19 pandemic by instigating major changes to service delivery. Immunisations have continued, although the number of vaccines delivered initially dropped in April 2020.

**Aim:**

To evaluate how COVID-19 impacted the delivery of immunisations in London and identify innovative practices to inform future delivery, including for COVID-19 vaccines.

**Design & setting:**

A mixed-methods study of immunisation delivery in London, UK.

**Method:**

An online survey of London general practices was undertaken in May 2020 to produce a descriptive analysis of childhood immunisation delivery and identify innovative delivery models. Semi-structured interviews were conducted between August and November 2020 to explore innovative immunisation models, which were analysed thematically.

**Results:**

Sixty-eight per cent (*n* = 830) of London practices completed the survey and 97% reported having continued childhood immunisation delivery. Common delivery adaptations included spaced-out appointments, calling parents beforehand, and having only one parent attend. Forty-three practices were identified as having innovative models, such as delivering immunisations outside practice buildings or offering drive-through services. The thematic analysis of 14 semi-structured interviews found that, alongside adaptations to immunisation delivery within practices, existing local networks collaborated to establish new immunisation delivery models. Local population characteristics affected delivery and provide insights for large-scale vaccine deployment.

**Conclusion:**

Immunisations continued during 2020 with practices adapting existing services. New delivery models were developed by building on existing local knowledge, experiences, and networks. Immunisation delivery during the pandemic, including for COVID-19 vaccines, should be tailored to local population needs by building on primary care immunisation expertise.

## How this fits in

Although the number of vaccines delivered initially decreased in April 2020, general practices in England continued providing immunisations during the 2020 COVID-19 pandemic. This study found that general practices across London continued to provide immunisations by making adaptations to how they were delivered within practices. Some general practices introduced innovative delivery models such as immunisations being given outside or via mass drive-through services. Innovative immunisation delivery was based on general practices’ existing local networks and expertise, which may support the implementation of other immunisation programmes such as COVID-19 vaccination.

## Introduction

The COVID-19 pandemic affected the delivery of immunisation programmes globally.^1–3^ As of May 2020, the World Health Organization found that 75% of countries surveyed were experiencing disruptions to immunisation programmes owing to COVID-19.^4^ Immediately following the implementation of the national lockdown in March 2020, the number of routine Measles, Mumps, and Rubella (MMR) vaccinations delivered decreased by 19.8% in England,^1^ before recovering over the following months.^5,6^ This disruption is likely to have arisen from both barriers to normal delivery within practices and confusion among patients about the availability of health services. Barriers within practices may have included the need to implement social distancing and adhere to infection prevention procedures, securing appropriate personal protective equipment (PPE), as well as staff availability and capacity. Patients may not have attended practices because they feared contracting COVID-19, faced transport barriers, or because of shielding and/or isolation requirements.^7^ It has been reported that parents were confused about whether scheduled immunisations were operating as usual^8^ and the government recommendation to ‘stay at home’ may have been interpreted as not needing to attend for immunisation appointments.^9^

International and national guidance was clear that routine immunisation services should continue during the COVID-19 pandemic and general practices in England were directed to prioritise the childhood immunisation programme, pertussis vaccination in pregnancy, hepatitis B vaccination to at-risk infants, and pneumococcal vaccination in at-risk groups.^10–12^ To continue delivering essential services, general practices had to make rapid changes to how care was delivered,^13^ including adaptations to routine immunisation delivery.^14^

Some innovative immunisation delivery, such as a practice in East London that offered a drive-through immunisation service, has been described.^15,16^ However, there is a lack of literature reporting on innovative immunisation delivery during the COVID-19 pandemic. First, this study aimed to understand how general practices in London adapted their delivery of routine childhood immunisations to maintain population protection against vaccine preventable diseases during the COVID-19 pandemic. Second, the study examined how practice adaptations and innovative delivery models could support future routine immunisation services, including COVID-19 vaccination programmes.^17^

## Method

This study utilised a mixed-methods approach. All London-based general practices (referred to as ‘practices’ hereafter) were invited to complete an online survey to provide a descriptive analysis of immunisation delivery in London during the pandemic. The survey findings identified different models of innovative practice, which were explored in more depth through qualitative semi-structured interviews.

### Online survey of London GP practices

An online questionnaire was developed and emailed to all 1215 London practices via a Public Health England Select Survey link on the 28 April 2020. The questionnaire consisted of six questions and asked practices whether they had delivered childhood (0–5 years) immunisations in the last 7 days, the adaptations they had made to their delivery of childhood immunisations, and what support they needed to continue to be able to vaccinate their eligible 0–5 years population (Supplementary Box S1). Practices were asked to complete the questionnaire by 29 May 2020. All practices were sent a reminder email a week before this final deadline. Contact with the practices was facilitated by the NHS England/Improvement (London Region) Immunisation Commissioning Team, who also emailed reminders to the clinical commissioning group (CCG) areas with lower response rates before the deadline.

### Analysis of survey data

Questionnaire data were exported from the Public Health England (PHE) select survey portal into Microsoft Excel for analysis. A descriptive analysis of the survey in Excel was considered appropriate to identify case studies for further exploration.^18^ Duplicate responses were removed, and responses coded into categorical and continuous variables, in accordance with an agreed codebook. The open-ended questions on how immunisations were being delivered and the support they needed were post-coded into nominal variables. Two researchers (HS and CH) independently identified response categories and compared findings. Frequency and percentage distributions were used to summarise survey responses. Forty-three practices were identified as having innovative models of immunisation delivery.

### Semi-structured interviews

The purpose of the interviews was to examine the adaptations and new immunisation delivery models developed by practices. In the sampling adaptations within practices and new delivery models were differentiated (Box 1) and practices allocated accordingly.

Box 1 **Adaptations to immunisation delivery within practices and new immunisation delivery models**

**Adaptations to immunisation delivery within practicesincluded the following:** spaced-out appointments; one parent only attending appointments; pre-calling or triaging patients before they attend appointments; staff wearing personal protective equipment (PPE); changes to the patient flow system; face coverings being worn by parents; having a designated room at the practice; ‘red book’ immunisation recording changes; active call and recall; and immunisations newly being delivered as part of the 6–8 weeks check.

**New immunisation delivery modelsincluded the following**: drive-through or walk-in immunisation clinics in GP car parks; or using non-health infrastructures (such as football stadiums) to host immunisation sessions (only flu in the present study); separate delivery via cold and hot hubs arranged by GP federation, primary care network, or clinical commissioning group (CCG), with hot hubs managing patients with suspected COVID-19 symptoms and cold hubs non-COVID-19 suspected patients.

A maximum variation purposive sampling approach was applied^19^ to recruit practices from different geographies in London. This approach involved two stages: (i) The NHS England/Improvement (London Region) Immunisation Commissioning Team emailed a study information letter on 9 August 2020 to the 43 practices that reported using new delivery models in the survey, inviting them to participate in a semi-structured interview; (ii) application of a more strategic approach to ensure that the sample included practices that had used site-based and non-health infrastructure drive-through and/or walk-in models, and those that had used cold or hot hubs. This reduced the sampling frame to 34 London practices. These practices received a second email invitation asking them to contact the research team if they wanted to participate. If no response was received within a week, the research team called these practices to elicit interest in participation.

One practice was recruited via the first-stage and 10 via the second-stage approach. Three additional interviews were conducted; two with CCG representatives as a result of snowball sampling during two interviews, and one with a practice that was participating in non-health infrastructure mass flu immunisation events, which was identified via a professional network.

### Data collection

The interviews were conducted by five members of the research team (AH, CF, HS, SMJ, TC), who were independent from the management of the London immunisation programme. One interview was conducted in person, the others were conducted via telephone and online communication platforms. All interviewees were given the opportunity to ask questions before they signed an informed consent form and recorded an audio-recorded consent statement. The consent process included an explanation on how their confidentiality would be protected.

The purpose of the interviews was to gain insights into pre-defined topics (Supplementary Box S2) — for example, immunisation adaptations following the COVID-19 pandemic; interviewees’ perspectives on advantages and challenges of new adaptations and models; and views on their ongoing role in the routine immunisation programme and for the delivery of COVID-19 vaccines — while remaining attuned to other relevant content interviewees wanted to share. The interviews lasted between 20 and 60 minutes, and were conducted between 25 August and 10 November 2020.

### Data analysis

The interviews were audio-recorded with participants’ permission, anonymised, and transcribed verbatim by an external company. Transcripts were downloaded into a qualitative data analysis management programme (NVivo version 12). A thematic analytical approach was adopted that combined a semi-deductive coding of data to pre-identified topic areas and inductive interpretation to define overarching themes.^20^ All five interviewers pre-coded their interviews to develop a coding framework that was applied by SMJ and TC to the whole dataset. The whole team reviewed the dataset, verified the coding, and engaged in interpreting meanings and grouping codes under the overarching themes.

## Results

### Online survey of London general practices

The survey response rate was 68% (*n* = 830/1215) among all London practices. It varied across London, from 87% of practices in north-east London to 44% of practices in south-east London. Ninety-seven per cent (*n* = 805) of practices had provided routine childhood (<5 years) immunisation appointments in the previous 7 days. Among the 25 practices that reported not delivering, 52% (*n* = 13/25) had not stopped immunisation appointments, but either had no registered children due immunisations or were referring them elsewhere for immunisation. Six per cent of practices surveyed (*n* = 47) reported that they had issues with some patients cancelling or not coming in.

Twelve different adaptations to routine immunisation delivery were identified. The most common adaptations mentioned by responders were ‘spaced-out appointments’ followed by ‘one parent only’ attending. Practices also commonly reported that they were pre-calling patients before they attended their immunisation appointments and that staff were wearing PPE to deliver immunisations. Other less frequently mentioned adaptations included: operating a patient flow system; face coverings being worn by parents; having a designated room at the practice; the parent-held record of immunisations (the ’red book’)^21^ not being completed at the time of the appointment but parents receiving a print-out to add to the book at home; waiting-room adaptations; active call and recall; and immunisations being delivered as part of the 6–8-week baby check (which, while standard at some practices before the pandemic, was reported as an adaptation by some responders). Innovative models of delivery were mentioned by 5% of practices (*n* = 43). These consisted of immunisations being administered outside, drive-through or walk-through models, immunisations being delivered at separate sites, and collaborations with primary care networks (PCNs) or general practice federations. The most common support that practices wanted were PPE supplies and public health campaigns to promote vaccination during the COVID-19 pandemic, which were both mentioned by just under one-fifth of practices. Fewer responders requested support for access to COVID-19 staff testing, outreach for vulnerable patient groups, or access to cold hubs for patient referrals and additional staff.

Initial results of the survey were shared with stakeholders across the system and prompted initiatives such as training for vaccinators in delivering immunisations safely during the COVID-19 pandemic.

### Semi-structured interviews

Fourteen semi-structured interviews were conducted with representatives from 12 practices and two CCGs in London (see Table 1 for breakdown by CCG and sustainability and transformation partnership areas).^22^ The immunisation uptake in the 12 practices was above average for London.^23^ The thematic analysis identified five overarching themes: ‘Demonstrating and communicating the safety of immunisation services during the pandemic’; ‘Innovation aimed at enabling vaccine delivery’; ‘The benefit of pre-existing collaborative practice’; ‘Existing knowledge of local population’; and ‘Lessons for large scale vaccine deployment’.

**Table 1.** Semi-structured interviews: location and interviewee characteristics

**Interview #**	**Site**	**Role of interviewee(s**)	**STP area**	**Date of interview**
1	General practice	Practice nurse	North east London	15/09/2020
2	General practice	Business manager	North west London	17/09/2020
3	General practice	Practice manager	South west London	22/09/2020
4	General practice	Practice nurse	North central London	22/09/2020
5	General practice	GP	North central London	01/10/2020
6	CCG	i. GP collaboration ii. CCG flu team member	South west London	07/10/2020
7	General practice	Practice nurse	North London	10/11/2020
8	General practice	Practice nurse	South east London	25/08/2020
9	General practice	Practice nurse	North east London	07/09/2020
10	General practice	Deputy practice manager	North east London	11/09/2020
11	General practice	Assistant practice manager	North east London	14/09/2020
12	General practice	Business manager	North west London	22/09/2020
13	General practice	Practice manager	North central London	24/09/2020
14	CCG	Training	South east London	06/10/2020

CCG = clinical commissioning group. STP = sustainability and transformation partnership (an area where local NHS organisations and councils have drawn up shared proposals to improve health and care in the area they serve).^22^

#### Demonstrating and communicating the safety of immunisation services during the pandemic

The purpose of the adaptations introduced by practices (Table 2) was to demonstrate and communicate that immunisation clinics were *'COVID safe'* (Interview 2)*,* so that patients would not be deterred from attending. Many made changes similar to those reported by the practices surveyed in April–May 2020, with additional emphasis placed on ensuring that sites adhered with socially distancing and infection control measures. Interviewees also elaborated on additional patient engagement before and during vaccine appointments.

**Table 2.** New delivery models and adaptations to practices used by different practices

Interview number	New delivery models	Collaborative practice	Adaptations to practice
Outside delivery at GP site (including drive through)	Off-site (drive through or walk-in at non-health building)	Cold and hot hubs	Site or building adaptations (waiting area, logistics)	Administrative adaptations (appointments, call and recall, staff changes)	Safety or infection control adaptations
GP 1	*√*					**√**	**√**
GP 2	*√*					**√**	**√**
GP 3		*√*		*√*	**√**	**√**	**√**
GP 4	*√*				**√**	**√**	**√**
GP 5 (multiple practices)	**√**		**√**	**√**	**√**	**√**	**√**
CCG 6		**√**		**√**	**√**	**√**	**√**
GP 7		**√**	**√**	**√**		**√**	**√**
GP 8			**√**	**√**		**√**	**√**
GP 9			**√**	**√**		**√**	**√**
GP 10			**√**	**√**	**√**	**√**	**√**
GP 11			**√**	**√**	**√**		**√**
GP 12			**√**	**√**		**√**	**√**
GP 13					**√**	**√**	**√**
CCG 14				**√**			

CCG = clinical commissioning group

Practices’ ability to create safe one-way immunisation pathways depended significantly on the layout of their site, the number of access points, and existing structures. Some practices could section off internal cold-hub areas; others developed new delivery models involving external spaces or collaborating with other practices:

*'So, there’s one side of the building that’s got a fire escape and they come in through the main entrance, they come up the stairs to see the nurses which is called the “cold hub” and then they go out via the fire escape.'* (Interview 8)

Interviewees received ample information from PHE, NHS England (NHSE), and CCGs about how to deliver safe immunisation services, but found the frequent changes in guidance challenging:

*'… you kind of felt like you were constantly trying to read up, and then everyone had a, “Oh, I think I’ve seen this”, or, “Did you read that?” so that was kind of exhausting trying to keep up with, are we still doing what we’re meant to be doing …?*' (Interview 2)

This second-guessing could inadvertently undermine staff’s confidence in communicating clear messages and being consistent in the implementation of infection control protocols. A *'confident and consistent'* (Interview 12) approach was viewed as essential for keeping practices COVID-safe and preserving public trust.

One interviewee related how when their *'front door was actually closed, and we couldn’t just let anyone in without reason … we had to call more patients than what we’d normally have to do'* (Interview 13). For childhood immunisation, this involved phoning parents to pre-book appointments and reassure them that it was safe to come to the surgery. Practice staff likened these calls to *'telemedicine*' (Interview 8) or *'pre-consultations'* (Interview 3) as they provided the opportunity to provide practical explanations and address wider concerns about immunisation.

One interviewee relayed how, although very time-consuming (*'… we had to work really, really hard … a day or so a week just ringing parents … to encourage them to bring their children in…',* [Interview 13]), this type of telephonic dialogue achieved the *‘* *biggest impact*’ in addressing fear about accessing practices during the lockdown (Interview 13). This engagement helped nurses talk to parents who had delayed immunisations for other reasons, and practice staff used lockdown to work through their lists of children needing immunisation recalls.

Practices added more appointments to their schedules during lockdown and increased their duration, in many cases increasing the time allocated (for example, from 15 to 30 minutes or 30 to 45 minutes). Extending appointments facilitated adherence with infection control measures during and between appointments, created space for more interaction, and enabled combining childhood development checks with immunisations, thereby making the most of every contact and reducing risk of exposure to COVID-19.

#### Innovation aimed at enabling vaccine delivery

Table 3 summarises the new delivery models developed in London, citing their characteristics, strengths, and weaknesses. These models were borne out of the need to address the impact of the lockdown on falling immunisation rates and the desire to offer patients a choice in how they could access vaccines. As Interviewee 8 said, *'People were so frightened to come to the surgery.'*

**Table 3.** Characteristics, strengths, and weaknesses of new delivery models and adaptations to practice

Model	Characteristics	Strengths	Weaknesses
At GP site	Drive-through	Immunisations given within vehicle by nurse in practice car park, often through window or car door.	Whole family can be immunised at once when needed Reduced contact risk by not entering practice Patients reassured by reduced contact risk Efficient and short appointment times	Patients need own vehicle Practice needs appropriate car-park space Additional staff needed to support vaccinator with equipment, such as sharps bins May impede the ability to monitor for post-vaccination anaphylaxis
	Outside walk-in	Immunisations given by practice nurse outside under tent or gazebo cover.	Reduced contact risk by not entering practice Patients reassured by reduced contact risk Efficient and short appointment times	Weather-dependent, temperature risk for small children Reduced contact means there may be less opportunity for parents to ask opportunistic questions and for staff to reassure children and families Multiple staff may be required outside to support vaccinator with equipment, such as sharps bins
Off-site delivery in non-health buildings	Drive-through and walk-in	Immunisations given by practice nurse outside at alternative site, such as retail car park or sports ground.May be drive-through, walk-in or both.Used for mass flu vaccination campaigns.	Reduced contact by not entering practice Accessible site with public transport links Efficient and short appointment times Patients reassured by reduced contact risk May still be able to see ‘own’ or familiar practice staff	Patients may need own vehicle, if drive-through site May be further to travel from usual practice May only offer to certain groups, such as aged >18 years, <80 years Weather-dependent, if uncovered Additional staff needed to support vaccinator with administrative tasks and equipment, such as sharps bins May not be able to see ‘own’ practice staff and may miss opportunities for holistic assessment of children and families, and detection of wider health issues such as postnatal depression May impede the ability to monitor for post-vaccination anaphylaxis
General practices designated as hot or cold hubs		General practices designated as hot (red) hubs — only for patients with COVID-type symptoms, or cold (green) hubs — for all other work, including immunisations. Decision making at GP federation, primary care network, or CCG level	Safety of keeping all potential COVID-19 cases away from at-risk patients Patients reassured by reduced contact risk	May be further to travel from usual practice Health professionals less familiar with families may be less able to identify and follow up safeguarding concerns Some practices may not benefit, owing to different IT systems

CCG = clinical commissioning group

The design and iterative evolution of on-site models required teamwork:

*'we did like a mind map, there was myself, the lead GP, our admin team, practice manager … sat down and we kind of just made a little plan of how we could work around the premises and what we could do'* (Interview 1)

Similarly, pre-existing collaboration between the GP federations, PCNs, and CCGs meant that practices could be designated and set up as cold and hot hubs within weeks of the start of the first lockdown. This collaboration also supported the transfer of staff across networks to provide cover needed owing to staff illness or shielding, particularly at the beginning of the pandemic.

The use of the on-site models was contingent on access to appropriate outdoor space, and all walk-in and drive-through models were subject to risk assessments. The off-site models were established at a later stage of the pandemic to cater for the expanded flu vaccination campaign.

All models required staff to adapt to new ways of working and, in some cases, new premises. The new models also required additional human and material resources. Flexibility was required throughout, with continual planning and adjustments made to the models in response to experiential learning. New models were generally well-received by patients, especially if they were conducted by familiar practice staff, offered efficient and streamlined appointments, and did not require them to travel long distances to access immunisations. With reference to a GP site drive-through, one interviewee stated:

*'... they’ve actually loved it. It’s surprising because initially we weren’t sure whether it would work … but because now that they’ve been quite used to the idea … with the pandemic everything has changed. So, this is the norm now.'* (Interview 1)

#### The benefit of pre-existing collaborative practice

*'Working at scale'* as a fall-back position was presented by one interviewee (Interview 12) as core to the public health agenda early in the pandemic. Several interviewees highlighted that this approach built on the pre-existing PCN strategy of supporting partnership working in primary care.

Practices that were part of well-functioning collaborative networks used these to help them catch up on routine immunisations post-lockdown in the following manner: (i) GP federations operated booking systems that enabled patients to book appointments at different practices, and ran extended access clinics across their network; (ii) to create cold and hot hubs; (iii) to access off-site immunisation events organised by CCGs or PCNs.

Collaboration was not without its challenges and could result in smaller practices feeling they were being driven by targets or goals that did not suit them. Concerns were also raised about maintaining close relationships with patients in larger models. Two practice nurses also described the support they gained from practice nurse forums and questioned whether forum leads were sufficiently involved in the pandemic response.

#### Existing knowledge of local population

Interviewees referred to population groups whose access to immunisation was impeded by their living arrangement, language, or underlying vaccine hesitancy. Immunisation awareness among populations with English as a second language was described as limited:

*'English isn't the first language of the majority of our patients. So, they’re not really aware of the immunisation schedule.'* (Interview 13)

The transiency of some populations was also perceived to create access barriers:

*'We’ve got a very transient population and in the local area of theprimarycarenetwork, one of the practices is just for homeless people* [people experiencing homelessness] *...* [and] *One of the practices* *…* [has high numbers of] *university* [students] *...* *we have to take that into consideration* *.'* (Interview 12)

Several practices relayed their experience of addressing vaccine hesitancy among local ethnic minority populations. One intervention used for this purpose involved connecting parents with a *‘* *s* *ocial* *p* *rescribing* *l* *ink* *w* *orker* *’* who seeks to engage underserved communities (Interview 5). Practice staff also highlighted that many families did not want to travel long distances: *'They’d have preferred to come to …. their local surgery I think’* (Interview 8). Local knowledge also extended to the logistics of newer delivery models, such as mass flu vaccination sites being on *'a very busy road; people assume there’s going to be a lot of traffic … so there is just this resistance and they would rather go to their pharmacy than come'* (Interview 6).

#### Lessons for large-scale vaccine deployment

Interviewees suggested the new delivery models could be adapted and used for larger-scale vaccine programmes. Indeed, some practices built on the delivery adaptations they had instigated during the first lockdown (March–June 2020) to deliver their flu vaccine programme in autumn 2020:

*'The reason it worked quite easily is because we had done that in COVID. We’d been a green practice and they’d been a red practice. So, their GPs were used to working in our building. So, we just thought … Everything’s set up to do that again and have our flu clinics there so that we can have the whole building to do … socially-distanced flu clinics.'* (Interview 7)

Interviewees also discussed how flu vaccine delivery models could be used for the deployment of COVID-19 vaccines:

*'I think we would just do it* [COVID-19 vaccines] *in a similar way that we do flu … I think it can be delivered in general practice, but I think you have to have a system to do it. And I think the flu system fits with that.'* (Interview 2)

This included discussing the role of practices in achieving high uptake of COVID-19 vaccines:

'... *so I think if the delivery is through the GPs, that will lead to a good amount of uptake of a COVID-19 vaccine.'* (Interview 5)

Interviewees also drew attention to other limitations of off-site models: *'… it may be better as a mass flu clinic … but you might lose some of the trust a GP surgery would have'* (Interview 7). The same interviewee reported that their more diverse practice population were less likely to attend the mass off-site flu clinics. Interviewees also reported logistical challenges to delivering these models, such as the additional demands made on existing practice staff and how local factors, such as transport, affected access to sites away from patients’ own practices (Interviews 3, 6, and 7).

## Discussion

### Summary

The survey found that London practices continued to deliver routine childhood immunisations during the pandemic. They responded to the infection control challenges posed by COVID-19 through a range of adaptations such as offering spaced-out appointments; restricting to one parent accompanying children; and utilising pre-appointment triage to encourage attendance and pre-screen clinic attendees. It was found that a smaller number of practices (5% of those surveyed) reported more innovative models of delivery. To the authors' knowledge, this is the first study to report on COVID-19-related immunisation delivery adaptations in England. This study provides valuable insights into how London practices were delivering the national child immunisation programme during the early stages of the pandemic.

Interviewees reconfigured their practices to ensure safe delivery of vaccinations, continued engaging patients remotely, and some developed tailored infrastructure and partnerships to conduct mass vaccination. Unintended benefits of these adaptations included pre-appointment patient engagement and strengthening of collaboration between practices.

The London-based practices interviewed reported how their immunisation delivery evolved over the course of the pandemic according to their capacity, local infrastructure, and population needs. For example, practices utilised their experience of operating cold and hot hubs for childhood immunisations to deliver flu immunisations to their populations. The models required a sound knowledge of the registered population demographics and pre-existing collaboration across PCNs, GP federations, and CCGs. However, since this innovation required additional staff and in some cases investment, it remains to be seen whether these models are sustainable for routine childhood immunisation. Practices that used adapted forms of the models for the influenza vaccination programme, indicated that they could be useful platforms for delivering COVID-19 vaccines.

### Strengths and limitations

The combination of closed and open survey questions enabled how many practices were continuing to deliver immunisations to be quantified, and also gave responders the opportunity to elaborate on practice adaptations. One limitation was that it only captured adaptations being used in May 2020 and, as the qualitative interviews highlighted, the delivery of immunisations evolved over the course of the pandemic. The response to the survey was 68% of all 1215 practices in London. There is no longer an agreed appropriate rate for survey responses;^24^ nevertheless, a lower response rate was achieved from some parts of London, which affected the geographical representativeness of responses. For example, those who did not respond may be operating within areas that may not have the means to adapt their delivery of vaccination services or were disproportionately impacted by the lockdown. The survey used open-ended, free-text responses to enable it to act as a scoping survey to identify innovative practice; however, this may have meant that some responders did not mention all adaptations they were using, including more innovative ones. However, the questions enabled responders to provide detailed answers on adaptations, which facilitated the purposive sampling for the subsequent interviews.

There may be other models that the methodology did not identify, although the range of models provides evidence of initial lessons learnt on delivering vaccines in the COVID-19 context. A key limitation is, however, that the findings cannot be used to assess the impact of innovative models on immunisation uptake. ImmForm immunisation uptake data^23^ identified that the innovating practices interviewed had higher than the London average immunisation uptake, so they cannot be considered typical. This means it is unknown whether the use of innovative immunisation delivery models meant practices more successfully maintained immunisation uptake during the pandemic. The interviews did provide in-depth insights on how practices managed the constraints created by COVID-19, and how different innovative models operated.

### Comparison with existing literature

The findings of adaptations made to immunisation delivery are consistent with other literature on the changes made to primary care in England during the pandemic,^13,14,25^ and also adaptations in other countries, such as the US^26^ and Belgium^27^, in response to COVID-19. The delivery of vaccines in England dropped immediately following the March 2020 lockdown,^1^ similar to other countries.^2^ The circulation of clear guidance to maintain delivery^11,28,29^ resulted in the recovery of vaccination delivery over the following months.^5^ Local adaptations and additional communication with parents, described by the interviewees, are likely to have played an important role in curbing the decline in vaccination delivery. Similar adaptations have been reported to have helped to keep uptake rates stable in an area of Scotland during the COVID-19 pandemic.^30^

Although in May 2020 nearly one-fifth of London practices surveyed wanted public health campaigns to promote attending immunisation appointments, the findings showed that practices quickly overhauled their communication approach with parents by introducing tailored telephonic pre-consultation, which supported continuity of services and vaccination catch-up. Reflecting on how current immunisation delivery models could serve vaccine deployment during pandemics, practice staff raised issues also highlighted in the literature on immunisation delivery during the 2009 A/H1N1 influenza pandemic. In that pandemic, the use of pre-existing local vaccination systems promoted higher uptake of the H1N1 influenza vaccine because these health facilities and their staff were trusted by those targeted for vaccination.^31–35^ Mass vaccination models were reported to be logistically challenging and likely to exacerbate health inequalities since hard-to-reach groups were not proactive in seeking vaccination.^32^ This is consistent with the interviewees’ reports of user preference for receiving vaccinations at their practice rather than off-site, and highlights the importance of established patient and health professional trust, factors they emphasised were of particular importance in ensuring that vaccination services were accessible for minority ethnic groups.

### Implications for research and practice

Practices have demonstrated their capacity to adapt to significant constraints of the COVID-19 pandemic to continue delivering both childhood and influenza immunisations, thereby demonstrating their central role within the immunisation programme. Some London practices developed and implemented innovative delivery models that built on a collaboration within and across practices and commissioning groups. Further research is needed to determine the sustainability of such models in delivering routine immunisation programmes and also their impact on uptake rates.

The findings support the central and coordinating role of general practice in implementing the vaccination programmes during pandemics as trusted local immunisation providers.^17^ This central role has been demonstrated by the successful rapid rollout of the COVID-19 vaccination programme by GPs and PCNs.^36,37^ Primary care spearheading the current COVID-19 vaccine delivery programme in England is supported by World Health Organization guidance that delivery should be ‘…*in line with countries’ specific health systems and context’* and *‘… leverage existing service delivery structures’*.^38^ In the UK, the Royal College of General Practitioner’s logistical guide on COVID-19 vaccine delivery also highlighted that general practices are ‘… *particularly well prepared for large-scale vaccination programmes, with the capacity to effectively reach large numbers of patients quickly* …’.^39^

A key part of delivering the COVID-19 vaccines involves communicating with the public and patients both nationally and individually to reassure them about the vaccines. GPs are crucial for addressing vaccine hesitancy, notably among some ethnic minority populations,^40,41^ as evidence from the COVID-19 vaccine programme suggests lower uptake among some ethnic groups.^42^ The COVID-19 pandemic has shown how misinformation^43^ can be rapidly shared via social media. How COVID-19 vaccine misinformation attitudes impact on vaccine acceptance for both existing vaccines, such as influenza,^44^ and routine childhood immunisations needs careful monitoring and clear public health communication.^45^ As trusted providers of immunisations with a good understanding of their local population, general practices are well placed to lead on individual patient communication.^46^

Immunisations during the COVID-19 pandemic were maintained by London practices through delivery adaptations and innovation. General practices’ existing expertise should be used to deliver future immunisation programmes, including the COVID-19 vaccinations, as they are best placed to tailor delivery to their local population needs. Further research on how innovative models impact immunisation uptake among different local population groups is warranted.
